# Cellular Metabolomics Revealed the Cytoprotection of Amentoflavone, a Natural Compound, in Lipopolysaccharide-Induced Injury of Human Umbilical Vein Endothelial Cells

**DOI:** 10.3390/ijms17091514

**Published:** 2016-09-09

**Authors:** Weifeng Yao, Hui Li, Qinan Liu, Ye Gao, Jin Dai, Beihua Bao, Li Zhang, Anwei Ding

**Affiliations:** 1 Jiangsu Collaborative Innovation Center of Chinese Medicinal Resources Industrialization, and National and Local Collaborative Engineering Center of Chinese Medicinal Resources Industrialization and Formulae Innovative Medicine, Nanjing University of Chinese Medicine, Nanjing 210023, China; yaowf@njucm.edu.cn (W.Y.); missyhui2012@163.com (H.L.); liuqinan0728@163.com (Q.L.); scotter01@163.com (B.B.); awding105@163.com (A.D.); 2 School of Pharmacy, Nanjing University of Chinese Medicine, Nanjing 210023, China; yegaohlgya@163.com; 3 Department of Pathology, University of Washington, Seattle, WA 98195, USA; daij@u.washington.edu

**Keywords:** amentoflavone, cytoprotection, cellular metabolomics, human umbilical vein endothelial cells, lipopolysaccharide

## Abstract

Amentoflavone is one of the important bioactive flavonoids in the ethylacetate extract of “Cebaiye”, which is a blood cooling and hematostatic herb in traditional Chinese medicine. The previous work in our group has demonstrated that the ethylacetate extract of Cebaiye has a notable antagonistic effect on the injury induced by lipopolysaccharide (LPS) to human umbilical vein endothelial cells (HUVECs). The present investigation was designed to assess the effects and possible mechanism of cytoprotection of amentoflavone via metabolomics. Ultra-performance liquid chromatography/quadrupole time of flight-mass spectrometry (UPLC/QTOF-MS) coupled with multivariate data analysis was used to characterize the variations in the metabolites of HUVECs in response to exposure to LPS and amentoflavone treatment. Seven putative metabolites (glycine, argininosuccinic acid, putrescine, ornithine, spermidine, 5-oxoproline and dihydrouracil) were discovered in cells incubated with LPS and/or amentoflavone. Functional pathway analysis uncovered that the changes of these metabolites related to various significant metabolic pathways (glutathione metabolism, arginine and proline metabolism, β-alanine metabolism and glycine, serine and threonine metabolism), which may explain the potential cytoprotection function of amentoflavone. These findings also demonstrate that cellular metabolomics through UPLC/QTOF-MS is a powerful tool for detecting variations in a range of intracellular compounds upon toxin and/or drug exposure.

## 1. Introduction

Metabolomic studies have shown an enormous capacity for the description of pathological states in humans [1], animals [2] and cells [3], as well as giving diagnostic knowledge and providing mechanistic insight into biochemical effects of drugs [4,5]. Cellular metabolomics can supply information reflecting alterations to metabolic pathways, biochemical reactions, and other cellular processes [6]. Recently, cellular metabolomics has been used to systematically investigate the small-molecule metabolites in specific cells [7] and mitochondria [8]. Small molecule metabolites, which belong to different chemical categories such as amino acids, organic acids, fatty acids, nucleosides (and their conjugates), carbohydrates, aldehydes and ketones, provide a large amount of information of a living system at different status. These small molecules often work in conjunction with specific enzymes or interact with other metabolites or proteins to influence cellular metabolic pathways. The metabolite information directly reflect phenotypic alterations in response to genetic or environment changes, including toxin or drug stimulation. In every aspect of cell function, metabolism is either directly or indirectly involved. Hence, cellular metabolomics can be used as an important research tool for cellular biochemistry.

**Figure 1:**
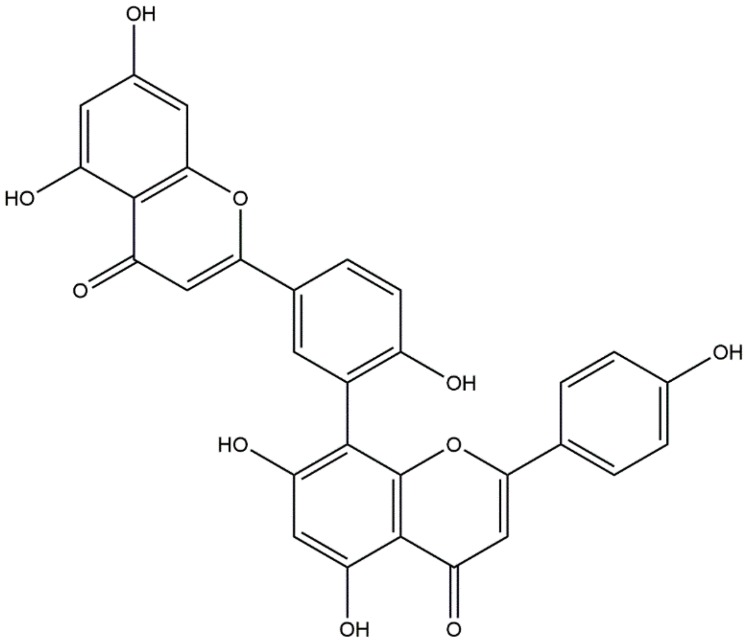
The structure of amentoflavone.

The Chinese herb, “Cebaiye” (dry branchlet and leaves of *Platycladus orientalis* (L.) Franco), is one of the commonly used herbal medicines in China [9]. It belongs to the blood “cooling” and hematostatic herb in the theory of traditional Chinese medicine [10]. Due to centuries of clinical application, it has been regarded as an effective herb for treating various symptoms, including hemorrhage in the interior or exterior of the body, chronic bronchitis and chin cough, tuberculosis, seborrheic alopecia and empyrosis. Our previous work has demonstrated that the ethylacetate extraction of Cebaiye has a notable antagonistic effect through mitigation of the damage induced by lipopolysaccharide (LPS) to human umbilical vein endothelial cells (HUVECs) [11]. The multiple flavonoids in the extract may be the active substances for protecting vascular endothelial cells [12], inhibiting cellular lipid peroxidation, and may decrease the production of nitric oxide (NO) [13]. Therefore, the bioactive flavonoids, e.g., amentoflavone, in Cebaiye should be further investigated for cytoprotective and hematostatic effects as well as the mechanism of action for cells [14]. Amentoflavone belongs to the biflavonoid class of flavonoids and has been used as an antioxidant, vasorelaxant, and anti-HIV agent [13,15,16,17]. The structure of amentoflavone is given in Figure 1.

To our knowledge, there has not been a comprehensive cellular metabolomic study on the cytoprotective effects of amentoflavone. In this work, an Ultra-performance liquid chromatography/quadrupole time of flight-mass spectrometry (UPLC/QTOF-MS) system in conjunction with multivariate data analysis is used to demonstrate the alterations of intracellular metabolite levels between untreated HUVECs and those incubated by LPS and/or amentoflavone. Some upregulated and downregulated molecules are observed in HUVECs, and further metabolic pathway analysis is discussed to enhance the understanding of the cytoprotective mechanism of amentoflavone.

## 2. Results and Discussion

### 2.1. Assay of NO, Malondialdehyde (MDA), and Superoxide Dismutase (SOD) Activity

LPS can influence endothelial cellular functions and morphology and open the cellular junction. As a result, it will aggravate vascular endothelial tissue permeation and lead to multiple organ failure [18,19]. In order to investigate the changes of anti-inflammatory and antioxidation under the influence of LPS and amentoflavone, the culture medium supernatant were evaluated by determining NO and MDA levels, and SOD activity (Table 1) [20,21,22]. The levels of NO and MDA in the model group were significantly higher than those in the vehicle control group (*p* < 0.01), but SOD activity was significantly lower (*p* < 0.01). The presence of amentoflavone with three different concentrations in treatment group were found to decrease the levels of NO and MDA and to increase SOD activity (*p* < 0.01, vs. model group). The reduction of NO in the amentoflavone treatment group indicates possible modulating effect on inflammation and in regulation of immune responses [23]. Nitric oxide synthase (NOS-2), a principal enzyme, could produce high-level and sustained NO. At high concentrations, NO generated by NOS-2 is oxidized to reactive nitrogen oxide species (RNOS) rapidly, which regulates most of the immunological effects [24]. RNOS can S-nitrosate the thiol group in glutathione (GSH) to produce S-nitrosoglutothione (GS-NO), which then acts as a NO and GSH reservoir. In mitochondrial respiration, some key enzymes are also prohibited by RNOS and this results in a depletion of cellular energy and ATP [25]. A conjunction of these interactions might explain the multiple functions of NO in the management of immune and inflammatory cells. Lipid peroxidation is a common event in toxic phenomenon. As a marker of lipid oxidation, MDA was analyzed to evaluate on LPS-induced oxidative stress [26]. SOD is one of the major antioxidant enzymes that can help to protect the body from oxidative damage [27]. Liu and coworkers have also demonstrated that the flavonoids treatment could reduce the NO and MDA content, and increase the SOD activity in LPS injured mice [28]. Thus, treatment with LPS caused an increase of NO and MDA and a decreased SOD activity compared with the vehicle control group, resembling the pathophysiological state of inflammation and oxidative stress. In the amentoflavone treatment group, the NO and MDA level decreased and SOD activity increased, suggesting that amentoflavone protects HUVECs against inflammatory and oxidative damage. Since the concentration of 18.587 μM could recover the three indexes most obviously, it was selected for further metabolomics study.

### 2.2. Multivariate Data Analysis

**Figure 2:**
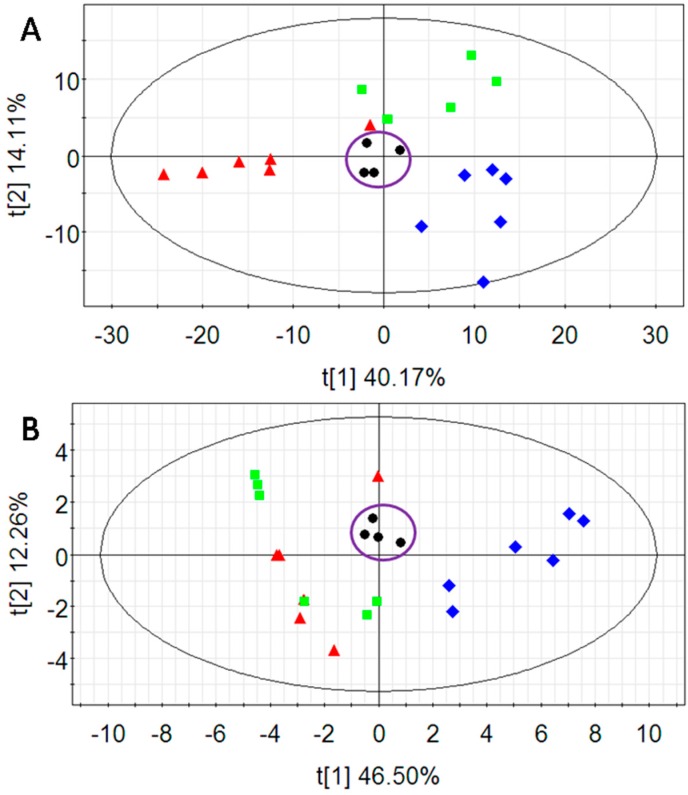
The score plots of principal component analysis (PCA): (**A**) positive, R^2^*X* = 0.618, Q^2^ = 0.396; and (**B**) negative, R^2^*X* = 0.749, Q^2^ = 0.248. Blue diamond: vehicle control group; Red triangle: model group; Green box: treatment group; Black dot: QC samples.

**Figure 3:**
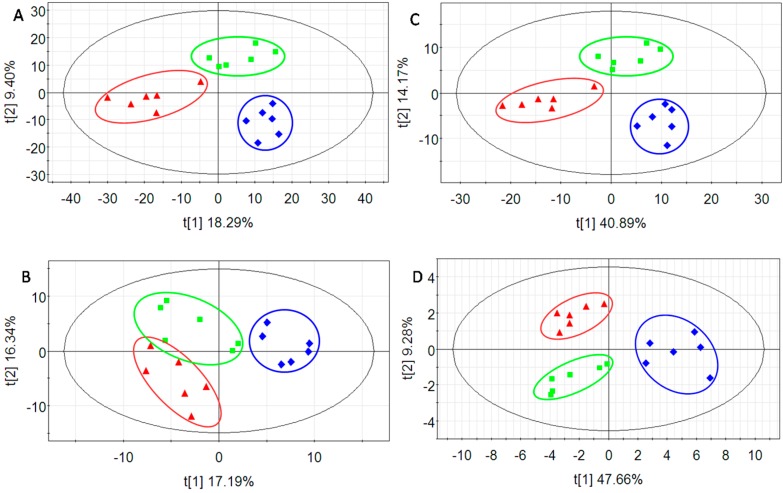
The score plot (*t*1/*t*2) of PLS-DA before (**A**,**B**) and after (**C**,**D**) ANOVA: (**A**) positive, R^2^*X* = 0.375, R^2^*Y* = 0.936, Q^2^*Y* = 0.579; (**B**) negative, R^2^*X* = 0.431, R^2^*Y* = 0.908, Q^2^*Y* = 0.617; (**C**) positive, R^2^*X* = 0.684, R^2^*Y* = 0.991, Q^2^*Y* = 0.843; and (**D**) negative, R^2^*X* = 0.569, R^2^*Y* = 0.831, Q^2^*Y* = 0.735. Blue diamond: vehicle control group; Red triangle: model group; Green box: treatment group.

First, principal component analysis (PCA), an unsupervised multivariate data analysis technique, was performed to visualize grouping trends and outliers in the data. The score plots of the first two principal components (*t*1/*t*2) of the data collected in positive and negative mode are shown in Figure 2A,B, respectively. The quality control (QC) samples clustered tightly in both score plots, indicating the stability of the analytical platform. To gain a better understanding of the metabolite difference among vehicle control, model and treatment groups and the efficacies of amentoflavone against cell injury, projections to latent structures discriminate analysis (PLS-DA) was used to construct a model where the variable matrix was made up of LC/MS ion peak areas of features (Figure 3). Ordinarily, the relevant R^2^*X* (the cumulative fraction of sum of squares of *X* explained by components), R^2^*Y* (the cumulative sum of squares of all the *y*-variables explained by the extracted components), and Q^2^*Y* (the fraction of the total variation of *Y* (PLS) that can be predicted by the extracted components) were used to evaluate the model quality of the PLS. It is unpractical to get a high Q^2^*Y* without a high R^2^*Y*. A Q^2^*Y* > 0.5 is considered as good and a Q^2^*Y* > 0.9 as outstanding [29], but these rules are heavily application dependent. If the differences between Q^2^*Y* and R^2^*Y* are larger than 0.2~0.3, it indicates the presence of many irrelevant factors or some outliers.

Analysis of variance (ANOVA) was used before PLS to select the variables with *p* value < 0.05 and help build a statistical model that is more reliable and easier to interpret. As expected, the values of R^2^*X*, R^2^*Y* and Q^2^*Y* (Table 2) were more acceptable for data sets collected in positive and negative ionization mode after ANOVA, indicating an excellent prediction. Compared to the results before ANOVA, the lower differences between R^2^*Y* and Q^2^*Y* also suggests a more stable and robust analysis. The performances of PLS-DA are shown in the score plots (Figure 3). For the data collected with negative ionization, the samples from the model group and treatment group did not separate and the borders among the three groups were not clear without use of ANOVA (Figure 3B). After eliminating the variables with *p* values > 0.05, the classification of samples was more obvious among the three groups (Figure 3D), and the samples in each group in Figure 3C appeared closer than those in Figure 3A, indicating that the differences were minimized between samples within each group. Therefore, a robust ANOVA-PLS discriminant model was set up to analyze the data sets.

### 2.3. Tentative Identification of the Biomarkers and Pathway Analysis

After building PLS-DA model, variable importance analysis is carried out as the key step before biomarker analysis. The variables of PLS-DA was screened with a VIP value larger than 1.0. Through further putative metabolite identification, seven potential biomarkers are selected and summarized in Table 3 with their corresponding retention time, *m*/*z*, formula, adduct ion, trends, and cellular location. Glycine, argininosuccinic acid, putrescine, ornithine, spermidine, 5-oxoproline and dihydrouracil from positive or negative ionization mode have responded to perturbation (up- or down-regulation) in model and treatment groups. These putative metabolites exist in mitochondria or cytoplasm of cells according to the Human Metabolome Database, and they could be considered as potential markers for further biological pathway analysis.

**Figure 4:**
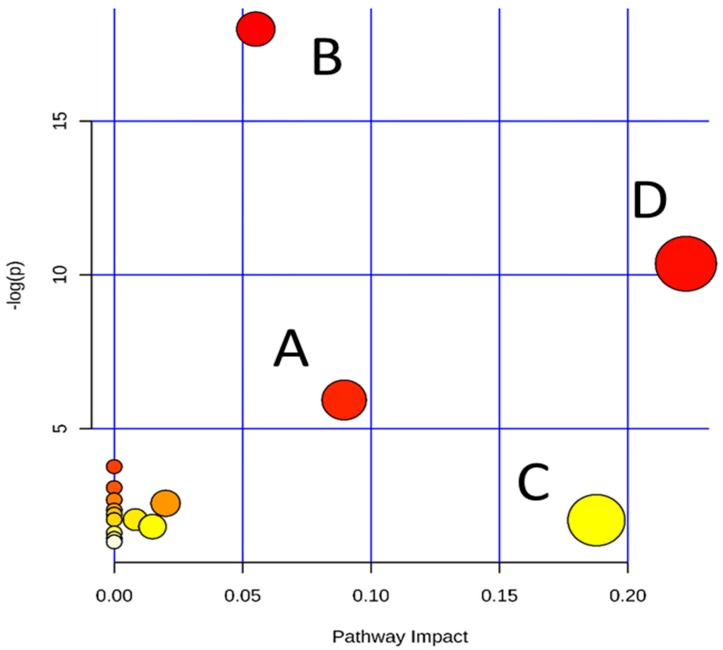
Overview of pathway analysis from pathway topology analysis. A: β-Alanine metabolism; B: glutathione metabolism; C: glycine, serine and threonine metabolism; and D: arginine and proline metabolism.

Metabolite profiling was often used to track the metabolites related to a specific metabolic pathway in biological status [30,31]. To determine whether the observed differences in the metabolites, in regards to cell injury, reflect coordinated changes in defined metabolic pathways, the pathway library of Human in MetPA software was used to identify the most relevant pathways involved in the conditions under evaluation. The MetPA assigned a total of 17 pathways for the feature compounds (Table S2). The dots in Figure 4 represent the pathways that were matched using pathway impact values from pathway topology analysis and *p* values from pathway enrichment analysis. Glutathione metabolism, arginine and proline metabolism, β-Alanine metabolism and glycine, serine and threonine metabolism, were revealed as the most important altered metabolic pathways. These important metabolic pathways were found to be affected in the cytoprotection of amentoflavone.

### 2.4. Effects of Amentoflavone on the Metabolic Pathway

**Figure 5:**
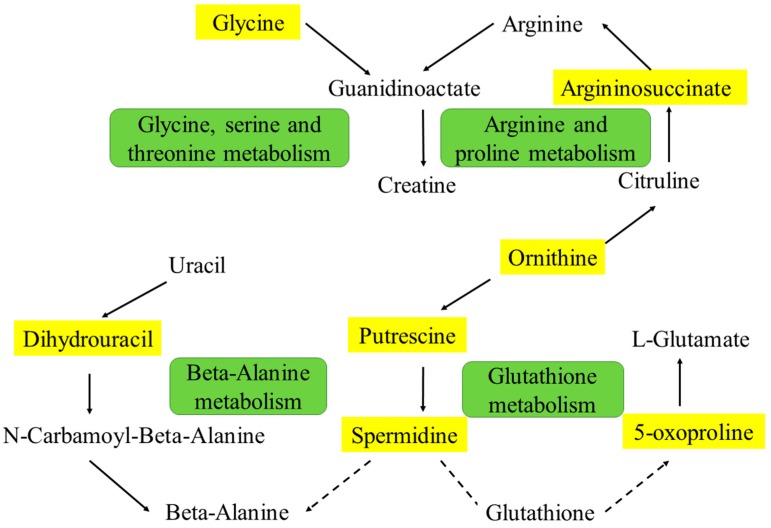
Correlation networks of all the potential biomarkers in LPS stressed and amentoflavone treated cells. Metabolites in yellow boxes represent altered biomarkers determined by UPLC/QTOF-MS. The green boxes represent the pathway including the metabolites around.

To understand the possible connections among these putative intracellular metabolites, we constructed the metabolic pathway map based on information obtained from the Kyoto Encyclopedia of Genes and Genomes Web site (www.genome.jp/kegg/), and the map is shown in Figure 5. In this study, putrescine, spermidine and 5-oxoproline could be correlated with glutathione metabolism. The levels of putrescine, spermidine and 5-oxoproline were decreased in cells of model group and elevated after the treatment of amentoflavone. Although GSH was not determined in intracellular extracts, it could be speculated that GSH might altered in the glutathione metabolism according to Figure 5. One possible explanation for this might be that GSH, the most abundant low-molecular-weight thiol in cells [32], plays important parts in nutrient metabolism, antioxidant defense, and regulation of cellular activities including cell apoptosis and immune response [33]. This possible explanation of metabolic pathway was identical with the discussion of biochemical assay results. The GSH level also reflected the alteration of the redox state, which is one of the key performance indicators in pathologic conditions [34,35]. The decreased levels of putrescine, spermidine and 5-oxoproline demonstrate that the cells in LPS-induced model group may be damaged, resulting in a reduction of glutathione levels. In the treatment group, the relative concentration of the three metabolites increased, indicating the capacity of the antioxidant, amentoflavone, to prevent damage from LPS [36].

Ornithine and argininosuccinate are basic amino acids in the urea cycle of arginine and proline metabolism [37]. In epithelial cells of the small intestine, citrulline and arginine are synthesized primarily using ornithine. Ornithine in liver cells surrounding the portal vein primarily acts as an intermediate of the urea cycle. In many peripheral tissues, ornithine is also supplied to synthesize glutamate and glutamine [38]. Some cells synthesize argininosuccinate from citrulline with argininosuccinate synthetase, and use it as a precursor for arginine in the urea cycle. Argininosuccinate is a precursor to fumarate in the citric acid cycle via argininosuccinate lyase [39]. Here, the levels of ornithine and argininosuccinate decreased in the model group and increased in the treatment group, demonstrating the cytoprotective effects of amentoflavone for injured HUVECs.

Dihydrouracil was also found to be disturbed in damaged HUVECs, which is an intermediate breakdown product of uracil in β-alanine metabolism [40]. β-alanine is formed in vivo by the degradation of dihydrouracil [41]. The increased level of dihydrouracil could lead to the high level of β-alanine, which will cause oxidative stress based on possible mechanism for the decreased enzymatic activity [42]. Glycine in Glycine, serine and threonine metabolism was given high score in MetPA, and the level is low in model group and increased in treatment group, showing the protection against oxidative stress in vascular tissue [43]. Weinberg and coworkers had also described that glycine is protective against cell death during oxidative injury induced by the addition of hydrogen peroxide to human endothelial cell [44].

## 3. Materials and Methods

### 3.1. Chemicals and Materials

The following chemicals and materials were obtained from the indicated suppliers: Acetonitrile (Merck, Darmstadt, Germany); formic acid (Merck, Darmstadt, Germany); Leucine encephalin (Sigma-Aldrich, St. Louis, MO, USA); Lipopolysaccharides (LPS; Sigma-Aldrich); Dulbecco’s modified Eagle’s medium (DMEM; Gibco, Grand Island, NY, USA); fetal bovine serum (FBS; Wisent, Saint-Jean-Baptiste, QC, Canada); trypsin (Biosharp, Hefei, China); Dimethyl sulfoxide (DMSO; Sigma-Aldrich); and Amentoflavone (Shunbo, Shanghai, China). Assay kits for malondialdehyde (MDA), superoxide dismutase (SOD) and NO were purchased from Nanjing Jiancheng Bioengineering Institute (Nanjing, China).

### 3.2. Preparation of Amentoflavone Solutions

The amentoflavone solutions of 4.647, 9.294 and 18.587 μM for treatment groups were prepared by diluting a 9.286 mM (5 mg·mL^−1^) solution of amentoflavone (in DMSO) with DMEM and filtered through a syringe filter with a 0.22 µm pore size hydrophilic polyethersulfone membrane (Florham Park, Morris, NJ, USA).

### 3.3. Cell Culture

HUVECs were purchased from Aiyan Biotechnology Company (Shanghai, China). The cells were cultured in DMEM containing 10% FBS, 100 mg·mL^−1^ penicillin, and 100 mg·mL^−1^ streptomycin at 37 °C in an atmosphere of 5% CO_2_ with the medium replaced every 24 h. After cell seeding and reaching 80% confluence (10 cm tissue culture dish), the cells from the model group were incubated with 100 μg·mL^−1^ LPS in DMEM for 24 h. For the treatment group, cells were incubated amentoflavone and 100 μg·mL^−1^ LPS in DMEM for 24 h. The cells in vehicle control group were given with an equal amount of DMEM. The concentration of LPS was determined from a survival rate test of HUVECs [11]. With 100 μg·mL^−1^ LPS, half of the total cells survived. Six replicates in separate dishes for each group were analyzed. Additionally, for testing the cytotoxicity of DMSO on HUVECs, a new experiment of the survival rate by MTT was carried out with different concentration and culture time (Table S1). The results showed that the survival rate of HUVECs with 0.2% DMSO is 98.12% ± 5.81% after culturing of 24 h, indicating that DMSO is compatible solvent vehicles towards the HUVECs.

### 3.4. Cell Metabolite Extraction

After incubation, the cells were harvested by addition of 0.05% trypsin/EDTA solution, washed with PBS and pelleted by centrifugation at 800 rpm for 6 min. The cell pellets were immediately dissolved in 1.0 mL cold mixture of methanol/water (4:1, *v*/*v*) at −20 °C [45]. Then the cells were ultrasonicated in an ice bath for 10 min and subsequently centrifuged at 13,000× *g* for 10 min at 4 °C. The supernatant was collected and dried with a stream of nitrogen. The residue was resuspended in 1.0 mL of acetonitrile/water (1:1, *v*/*v*), and then filtered through 0.22 μm mesh Millipore PVDF filters (Florham Park) into sample vials [46]. The samples were stored at −80 °C prior to analysis. In parallel, a quality control (QC) sample was prepared by mixing equal volumes of 30 µL from each of the 18 samples into a sample vial. The pooled QC samples were injected four times every 6 samples in order to monitor the stability of the analysis.

### 3.5. Biochemical Assays of SOD Activity, MDA Level and NO Level

HUVECs were suspended at a concentration of 1 × 10^5^ cells mL^−1^, seeded in 96-well microtiter plates (1 mL·well^−1^) and incubated at 37 °C in an atmosphere of 5% CO_2_ for 24 h. Then the cells in model group were incubated with LPS (100 μg·mL^−1^) in DMEM for 24 h. LPS (100 μg·mL^−1^) and amentoflavonewere both added in the treatment groups and the cells were cultured for 24 h. The amentoflavone concentrations of the three treatment groups were 4.647, 9.294 and 18.587 μM, separately. The cells in the vehicle control group were treated with an equal amount of DMEM. After an incubation period of 24 h for three groups, the supernatant of culture medium after centrifugation at 10,000× *g* for 10 min was used to measure SOD activity, MDA level and NO level with commercially available kits (Nanjing Jiancheng Bioengineering Institute, Nanjing, China) according to the manufacturer’s instruction. The obtained results were expressed as means ± S.E.M. The comparison between individual experimental groups was carried out with *t*-test and *p* values less than 0.01 were considered as significant differences.

### 3.6. UPLC/QTOF-MS Analysis of Intracellular Metabolites

UPLC analysis was performed on a Waters ACQUITY UPLC system (Waters Corporation, Milford, MA, USA) equipped with an Acquity UPLC BEH-C18 column (2.1 mm × 50 mm, 1.7 μm). The mobile phase was composed of A (100% acetonitrile), and B (0.1% formic acid in water, *v*/*v*) and the linear gradient was employed: 0–3 min, A: 5%–50%; 3–13 min, A: 50%–90%; 13–14 min, A: 90%; 14–15 min, A: 90%–5%. The flow rate of the mobile phase was held constant at 0.4 mL·min^−1^, and the column temperature was maintained at 35 °C. Mass spectrometry was carried out on a Synapt Q-TOF mass spectrometer (Waters, Manchester, UK). The instrument was operated using an electrospray source in both positive and negative mode. The ionization source conditions were as follows: capillary voltage of 3.0 kV, source temperature of 120 °C and desolvation temperature of 350 °C. The sampling cone voltage was set at 30 V, extraction cone was 2.0 V, trap collision energy was 6.0 V, transfer collision energy 4.0 V, trap gas flow was 1.50 mL·min^−1^, and ion energy was at 1.0 V. Nitrogen (99.99% purity) and argon (99.99% purity) were used as cone and collision gases, respectively. The cone and desolvation gas flow were 50 and 600 L·h^−1^, respectively. The scan time of 0.3 s and interval scan time of 0.02 s were used throughout, with a collision energy of 6 eV. The mass spectrometric data were collected from *m*/*z* 100 to 1000 in centroid mode. Leucine-enkephalin was used as the lock mass generating an [M + H]^+^ ion (*m*/*z* 556.2771) and [M − H]^−^ ion (*m*/*z* 554.2615) at a concentration of 200 pg·mL^−1^ and a flow rate of 100 μL·min^−1^. Dynamic range enhancement was applied throughout the MS experiment to ensure accurate mass measurement over a wider dynamic range. The typical total ion chromatograms of intracellular metabolites of normal, model and treatment groups were shown in Figures S1 and S2.

### 3.7. Multivariate Data Analysis and Data Processing

The raw UPLC/QTOF-MS data were first converted to NetCDF files by Databridge (Waters), and the files imported to the freely available software package MZmine 2.10 (http://mzmine.sourceforge.net/) for automated peak picking [47]. MZmine 2.10 was also used for peak noise removal, peak detection and alignment. The parameters of MZmine 2.10 in this study are listed in Table S3. The UPLC/QTOF-MS datasets were exported as an *m*-by-*n* matrices (*m*: the number of samples; and *n*: the number of ion peaks) containing values of ion peak area at different retention time and/or *m*/*z*.

Before multivariate data analysis, analysis of variance (ANOVA) was performed in Matlab (version 6.5, MathWorks, Natick, MA, USA) to eliminate variables with the *p* value > 0.05. The data set was then exported into SIMCA-P software 11.5 (Umetrics AB, Umeå, Sweden) for projections to latent structures discriminate analysis (PLS-DA). The unit variance (UV) scaling was selected to preprocess the data prior to PLS-DA and leave 1/7 out cross validation was used to determine the optimal model. The purpose of PLS-DA was to calculate models that differentiate between groups. In the PLS-DA modeling, the samples from different groups were sorted into different classes using score plots, and endogenous metabolites that contribute to the classification were found by variable importance in the projection (VIP) values, which showed the importance of each variable to the classification.

### 3.8. Biomarker Identification and Metabolic Pathway Analysis

For the identification of biomarkers, each spectrum was matched with the structure information of metabolites acquired from available biochemical databases, such as METLIN (http://www.metlin.scipps.edu/) [48], HMDB (http://www.hmdb.ca/) [49] and KEGG (http://www.genome.jp/kegg/) [50]. The pathway analysis of potential biomarkers was performed with MetPA software based on the pathway library of Homo sapiens (human) to identify the metabolic pathways. MetPA is now part of MetaboAnalyst (http://www.metaboanalyst.ca/MetaboAnalyst/) [51].

## 4. Conclusions

To our knowledge, this is the first cell metabolomic study exploring the cytoprotective function of amentoflavone. We reported the intracellular metabolic profile in HUVECs incubated with LPS and/or amentoflavone, using UPLC/QTOF-MS. Data analysis and preliminary compound identification revealed seven metabolites, glycine, argininosuccinic acid, putrescine, ornithine, spermidine, 5-oxoproline and dihydrouracil, which change significantly in cells incubated with LPS and/or amentoflavone. The seven metabolites might be tied to several important metabolic pathways, e.g., glutathione metabolism, arginine and proline metabolism, β-alanine metabolism and glycine, serine and threonine metabolism, which partially explain the cytoprotective properties of amentoflavone from a mechanistic point of view. Certainly, extra work, e.g., target metabolomics study [52], should be done for the confirmation of these preliminary metabolites and metabolic pathways. Taken together, our findings provided tentative evidence with a comprehensive metabolite profile on amentoflavone treatment of LPS-induced cell damage.

## Figures and Tables

**Table 1:** The protection of amentoflavone on injury of HUVECs induced by LPS (*n* = 5, $\bar{x}$ ± *s*).

Group	Concentration/μM	NO/μmol·L^−1^	MDA/nmol·mL^−1^	SOD/U·mL^−1^
Vehicle control	–	21.03 ± 0.86	1.71 ± 0.13	21.69 ± 2.16
Model	–	53.66 ± 3.07 *	2.80 ± 0.26 *	11.11 ± 1.20 *
Treatment	4.647	31.62 ± 3.01 ^#^	2.13 ± 0.13 ^#^	18.12 ± 0.50 ^#^
	9.294	27.33 ± 1.79 ^#^	1.93 ± 0.09 ^#^	19.26 ± 0.62 ^#^
	18.587	22.13 ± 2.28 ^#^	1.68 ± 0.17 ^#^	22.21 ± 1.67 ^#^

$\bar{x}$ represents the mean value and *s* represents the standard deviation; compared with vehicle control group, * *p* < 0.01; compared with model group, ^#^ *p* < 0.01

**Table 2:** The comparison of PLS-DA results.

Methods	Mode	Features	PLS-DA				
			A ^a^	R^2^*X*	R^2^*Y*	Q^2^*Y*	Difference ^b^
MZmine 2.10	Positive	1342	3	0.375	0.936	0.579	0.357
	Negative	241	3	0.431	0.908	0.617	0.291
ANOVA (*p* < 0.05) ^c^	Positive	307	5	0.684	0.991	0.843	0.148
	Negative	31	2	0.569	0.831	0.735	0.096

^a^ The number of latent variables; ^b^ The difference between R^2^Y and Q^2^Y; ^c^ The data from MZmine are processed by ANOVA before PLS-DA.

**Table 3:** Potential biomarkers and their cellular positions.

Mode	No.	R.T. (min) ^a^	Mass (*m*/*z*)	Adduct Ion	Delta	Formula	Compound	Trend ^b^	Trend ^c^	Cellular Location ^d^
Positive	1	0.343	113.9899	M + K	0.0053	C_2_H_5_NO_2_	Glycine	↓	↑	Mitochondria
	2	0.738	308.1522	M + NH_4_	0.0043	C_10_H_18_N_4_O_6_	Argininosuccinic acid	↓	↑	Cytoplasm
	3	4.988	127.0632	M + K	0.0000	C_4_H_12_N_2_	Putrescine	↓	↑	Mitochondria
	4	13.744	133.0936	M + H	0.0035	C_5_H_12_N_2_O_2_	Ornithine	↓	↑	Mitochondria
	5	14.671	163.1976	M + NH_4_	0.0058	C_7_H_19_N_3_	Spermidine	↓	↑	Cytoplasm
Negative	1	0.733	128.0383	M − H	0.0030	C_5_H_7_NO_3_	5-oxoproline	↓	↑	Cytoplasm
	2	14.850	113.0293	M − H	0.0063	C_4_H_6_N_2_O_2_	Dihydrouracil	↑	↓	Cytoplasm

^a^ R.T.: retention time of the components; ^b^ ↑ and ↓ represent up- and down-regulation of the metabolites in model group compared with the vehicle control group, respectively; ^c^ ↑ and ↓ represent the significant up- and down-regulation of the metabolites in amentoflavone treatment group compared with the model group, respectively; ^d^ The cellular position of metabolites from Human Metabolome Database (HMDB).

## Supplementary Materials

Supplementary materials can be found at www.mdpi.com/1422-0067/17/9/1513/s1.

## Abbreviations

LPS	lipopolysaccharide
HUVECs	human umbilical vein endothelial cells
NO	nitric oxide
NOS-2	type-2 isoform of nitric oxide synthase
RNOS	reactive nitrogen oxide species
GSH	glutathione
GS-NO	*S*-nitrosoglutothione
MDA	malondialdehyde
SOD	superoxide dismutase
PCA	principal component analysis
PLS-DA	projections to latent structures discriminate analysis
QC	quality control
UV	unit variance
VIP	variable importance in the projection
R^2^*X*	the cumulative fraction of sum of squares of *X* explained by components
R^2^*Y*	the cumulative sum of squares of all the *y*-variables explained by the extracted components
Q^2^*Y*	the fraction of the total variation of *Y* that can be predicted by the extracted components
ANOVA	analysis of variance
UPLC/QTOF-MS	Ultra-performance liquid chromatography/quadrupole time of flight-mass spectrometry
