# Supplementary material for: Cellular Metabolomics Revealed the Cytoprotection of Amentoflavone, a Natural Compound, in Lipopolysaccharide-Induced Injury of Human Umbilical Vein Endothelial Cells
Source: Int J Mol Sci. 2016 Sep 9;17(9):1514. doi: 10.3390/ijms17091514 (PMC5037791; doi:10.3390/ijms17091514)
Supplement: Supplementary file 1 [file ijms-17-01514-s001.pdf]

Supplementary Materials: Cellular Metabolomics Revealed the Cytoprotection of Amentoflavone, a Natural Compound, in Lipopolysaccharide-Induced Injury of Human Umbilical Vein Endothelial Cells

Weifeng Yao, Hui Li, Qinan Liu, Ye Gao, Jin Dai, Beihua Bao, Li Zhang and Anwei Ding

Table S1. The effect of different concentration and time on the survival rate of HUVECs ($\overline{X} \pm S$, $n = 6$).

Concentration (%)	12 h	24 h	36 h
0.05	97.25 ± 7.40	97.96 ± 4.65	82.56 ± 1.35
0.1	99.54 ± 3.76	97.18 ± 2.66	79.14 ± 2.58
0.2	98.12 ± 10.91	98.12 ± 5.81	77.41 ± 4.59
0.4	96.24 ± 4.04	94.61 ± 3.35	88.11 ± 5.47

$\overline{x}$ represents the mean value and *s* represents the standard deviation.

Table S2. Results from ingenuity pathway analysis with MetPA.

No.	Pathway Name	Total	Hits	Raw *p*	−log(*p*)	Impact
1	Glutathione metabolism	38	5	1.54×10^{-8}	17.991	0.05512
2	Arginine and proline metabolism	77	4	3.15×10^{-5}	10.364	0.22258
3	beta-Alanine metabolism	28	2	0.002644	5.9354	0.08953
4	D-Arginine and D-ornithine metabolism	8	1	0.023063	3.7695	0
5	Cyanoamino acid metabolism	16	1	0.045669	3.0863	0
6	Alanine, aspartate and glutamate metabolism	24	1	0.067825	2.6908	0
7	Thiamine metabolism	24	1	0.067825	2.6908	0
8	Pantothenate and CoA biosynthesis	27	1	0.076019	2.5768	0.02002
9	Methane metabolism	34	1	0.094899	2.3549	0
10	Nitrogen metabolism	39	1	0.10818	2.2239	0
11	Lysine degradation	47	1	0.12908	2.0473	0
12	Primary bile acid biosynthesis	47	1	0.12908	2.0473	0.00822
13	Glycine, serine and threonine metabolism	48	1	0.13167	2.0275	0.18774
14	Pyrimidine metabolism	60	1	0.16216	1.8192	0.01492
15	Aminoacyl-tRNA biosynthesis	75	1	0.19898	1.6146	0
16	Purine metabolism	92	1	0.23902	1.4312	0
17	Porphyrin and chlorophyll metabolism	104	1	0.26624	1.3234	0

Total is the total number of compounds in the pathway; the Hits is the actually matched number from the user uploaded data; the Impact is the pathway impact value calculated from pathway topology analysis.

Table S3. Parameter settings of MZmine 2.10.

Modules	Settings	Value
Centroid mass detector	Noise level	15
Chromatogram builder	Min time span (min)	0.03
	Min height	25
	m/*z* tolerance	0.04
Local minnimun search for Chromatogram deconvolution	Chromatographic threshold	65%
	Search minimum in RT range (min)	0.03
	Minimum relative height	5.0%
	Minimum absolute height	50
	Min ratio of peak top/edge	2
	Peak duration range (min)	0–2
Isotopic peaks grouper	*m*/*z* tolerance	0.02
	RT tolerance	0.01
	Maximum charge	2
	Representative isotope	Lowest *m*/*z*
Peak list rows filter	Minimum peaks in row	1
	Minimum peaks in an isotope pattern	1
	m/*z*	100–1000
	RT (min)	0–15
	Peak duration range (min)	0–2
RANSAC aligner	*m*/*z* tolerance	0.04
	RT tolerance	0.2
	RT tolerance after correction	0.05
	RANSAC iterations	10000
	Minimum number of points	30%
	Threshold value	0.03
	Linear model	Yes
Gap-filling	Intensity tolerance (%)	20
	m/*z* tolerance size	0.04
	RT tolerance size (s)	3
	RT correction	Yes

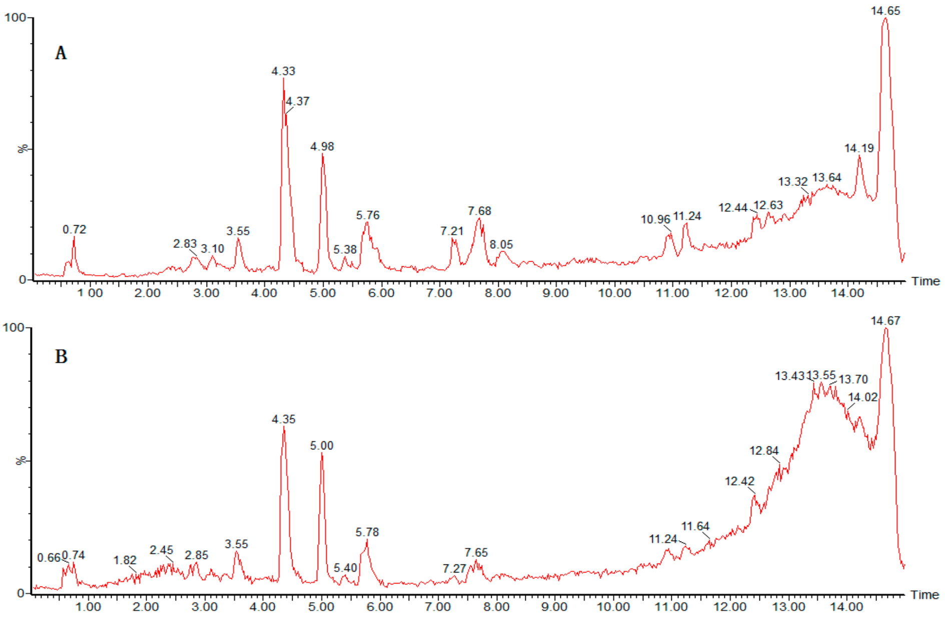

Figure S1. *Cont.*

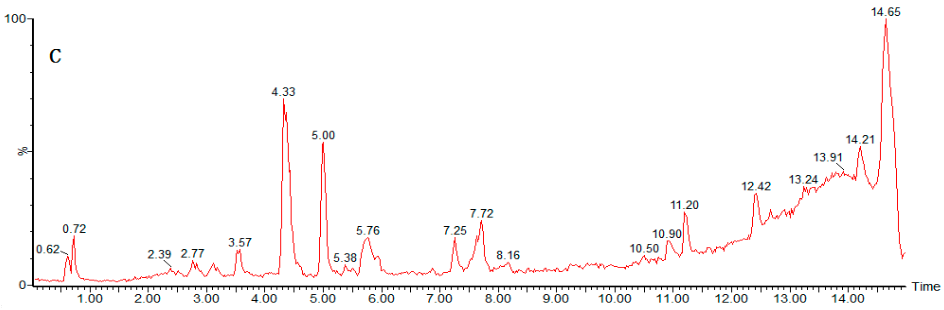

Figure S1. The base peak intensity chromatograms in positive mode. (**A**) Control; (**B**) Model; (**C**) Treatment.

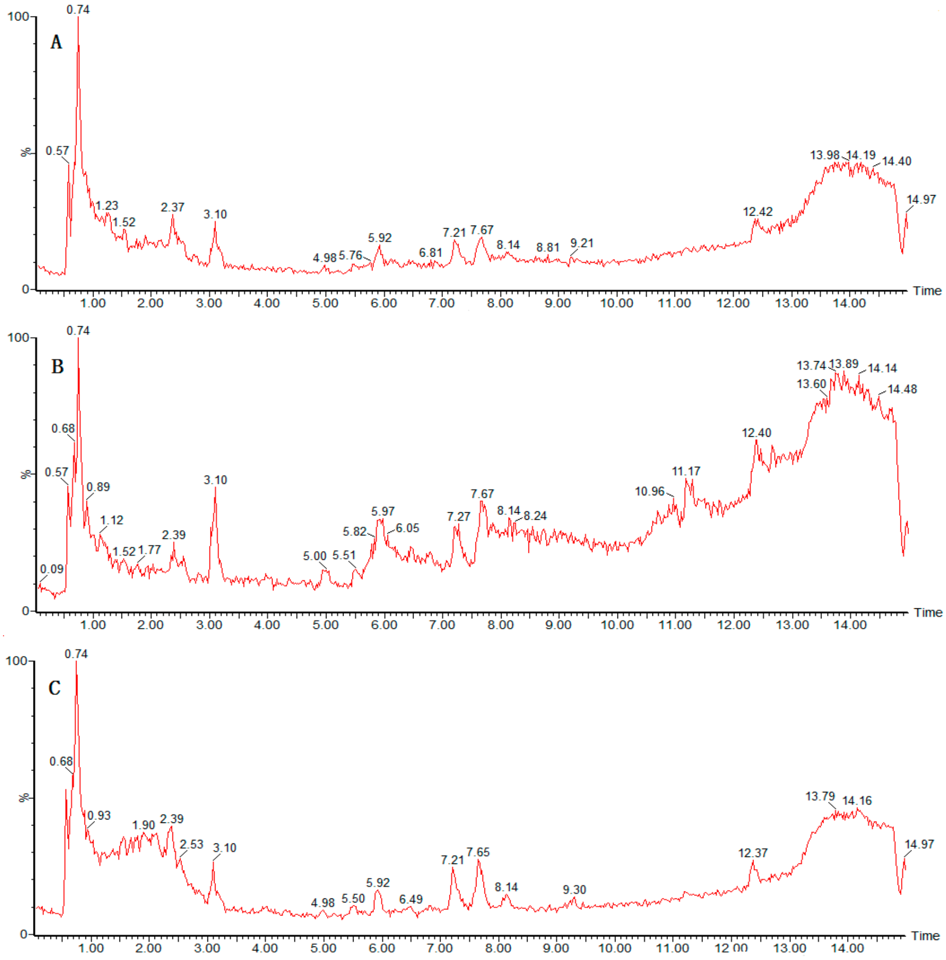

Figure S2. The base peak intensity chromatograms in negative mode. (**A**) Control; (**B**) Model; (**C**) Treatment.
